# Letter to the editor on: Perinatal ischemic stroke: a five-year retrospective study in a level-III maternity. einstein (São Paulo). 2015;13(1):65-71.

**DOI:** 10.1590/S1679-45082015CE3423

**Published:** 2015

**Authors:** Israel Macedo

**Affiliations:** Centro Hospitalar Lisboa Central, Lisboa, Portugal.

I read with interest the article by Machado et al.^1^ This work could be greatly improved by including an adequate control group, turning a merely descriptive case-series into a case-control study, with evaluation of odds ratios, thus giving a valuable contribution aligned with other recently published investigations.^2-5^ Some caution should be exercised when interpreting the incidence rates, since only symptomatic cases in the first 72 to 94 hours of life were included; whereas, according to the literature, some neonates present symptoms only after discharge and a few infants, within some months later.^4,5^ Data from the neonatal cohort registry would have more accurate estimate for incidence, like other studies.^3^ Considering our present level of knowledge in this field, is it ethical to spend investigators’, reviewers’, editors’ and readers’ time with vague research hypotheses and weak study designs?

## REPLY

Reply to letter to the editor on:Perinatal ischemic stroke: a five-year retrospective study in a level-III maternity. einstein (São Paulo). 2015;13(1):65-71.

Resposta a carta ao editor para: Acidente vascular cerebral isquêmico perinatal: estudo retrospectivo de 5 anos em maternidade nível III. einstein (São Paulo). 2015;13(1):65-71.

We thank Dr. Israel Macedo for his interest in our paper and comments that we will try to answer.

1. Inclusion of a control group

In our organization, it is not feasible to systematically screen stroke (by means of transcranial ultrasonography) in all newborns. Since stroke can be silent, and this was a retrospective study, we could have included patients presenting the disease in the control group, which would result in a selection bias.

2. Calculation of incidence rates

As explained in Methods, we reviewed cases of neonatal stroke in term newborns admitted to the neonatal intensive care unit. Other cases were out of our scope. Even if there were symptomatic cases after an uneventful hospital discharge, we would have no access to these patients. Our unit does not admit older patients and not all babies born in our organization return for pediatric follow-up visits.

Virgínia Machado, Sónia Pimentel, Filomena Pinto, José Nona

Maternidade Dr. Alfredo da Costa, Lisboa, Portugal.
