# Supplementary material for: Letter to the editor on: Perinatal ischemic stroke: a five-year retrospective study in a level-III maternity. einstein (São Paulo). 2015;13(1):65-71
Source: Einstein (Sao Paulo). 2015 Jul-Sep;13(3):478. doi: 10.1590/S1679-45082015CE3423 (PMC4943801; doi:10.1590/S1679-45082015CE3423)
Supplement: Translation

# Translation: Carta ao editor para: Acidente vascular cerebral isquêmico perinatal: estudo retrospectivo de 5 anos em maternidade nível III. einstein (São Paulo). 2015;13(1):65-71.

**Authors:** Israel Macedo

**Affiliations:** Centro Hospitalar Lisboa Central, Lisboa, Portugal.

Li com interesse o artigo de Machado et al.^1^ Este trabalho poderia ser melhorado se incluísse um grupo controle adequado, tornando uma mera série de casos descritiva em um estudo de caso-controle, com avaliação de *odds ratios*, e, assim, dando uma valiosa contribuição, alinhada a outras pesquisas publicadas recentemente.^2-5^ A interpretação de taxas de incidência deve ser feita com cautela, pois apenas os casos sintomáticos, nas primeiras 72 a 94 horas de vida, foram incluídos; enquanto alguns recém-nascidos apresentam/apresentaram sintomas apenas após a alta, e outros lactentes, depois de alguns meses.^4,5^Os dados do registro da coorte neonatal poderiam ter estimativas mais precisas para taxas de incidência, como em outros estudos.^3^ Se considerarmos o nível atual de conhecimento nesta área, é ético gastar o tempo de investigadores, revisores, editores e leitores com hipóteses de pesquisas vagas e desenhos de estudos tão fracos?

## RESPOSTA

Resposta a carta ao editor para: Acidente vascular cerebral isquêmico perinatal: estudo retrospectivo de 5 anos em maternidade nível III. einstein (São Paulo). 2015;13(1):65-71.

Reply to letter to the editor on: Perinatal ischemic stroke: a five-year retrospective study in a level-III maternity. einstein (São Paulo). 2015;13(1):65-71.

Agradecemos ao Dr. Israel Macedo por seu interesse em nosso artigo e pelos comentários, que procuraremos responder.

1. Inclusão de um grupo controle

Em nossa instituição, não é viável rastrear acidente vascular cerebral (AVC) de forma sistemática (por meio de ultrassonografia transcraniana), em todos recém-nascidos. Como o acidente vascular cerebral pode ser silencioso, e este foi um estudo retrospectivo, poderíamos ter incluído pacientes que apresentavam a doença no grupo controle, o que resultaria em um viés de seleção.

2. Cálculo das taxas de incidência

Como explicado na seção Métodos, fizemos a revisão dos casos de acidente vascular cerebral neonatal em recém-nascidos a termo, internados na unidade de cuidados intensivos neonatais. Outros casos fogem ao âmbito de nosso estudo. Mesmo se houvesse casos sintomáticos após uma alta hospitalar sem intercorrências, não teríamos acesso a esses pacientes, uma vez que nossa unidade não admite pacientes mais velhos provenientes do ambulatório, e nem todos os recém-nascidos são encaminhados para consultas de acompanhamento pediátrico após a alta.

Virgínia Machado, Sónia Pimentel, Filomena Pinto, José Nona

Maternidade Dr. Alfredo da Costa, Lisboa, Portugal.
